# Presentation of a keratocystic odontogenic tumor with agenesis: a case report

**DOI:** 10.1186/1752-1947-8-126

**Published:** 2014-04-09

**Authors:** Mariano Lacarbonara, Giuseppe Marzo, Vitantonio Lacarbonara, Annalisa Monaco, Mario Capogreco

**Affiliations:** 1 Department of Life, Health and Environmental Sciences, Dental Clinic, University of L’Aquila, Unit of Dentistry, Building Delta 6, Via Vetoio, 67100 L’Aquila, Italy; 2 Complex Operating Unit of Odontostomatology and Surgery, Interdisciplinary Department of Medicine, University of Bari, Piazza Umberto I, 1, 70121 Bari, Italy

**Keywords:** Odontogenic keratocyst, Keratocystic odontogenic tumor, Hypodontia, Lateral agenesis

## Abstract

**Introduction:**

We analyzed the etiopathogenetic, clinical, radiographic, and histopathologic aspects of keratocystic odontogenic tumors, particularly in association with dental anomalies of number, with the aim of providing useful information for their correct diagnosis, treatment, and prognosis within a multidisciplinary approach.

**Case presentation:**

A 14-year-old Caucasian girl presented for observation of bilateral agenesis of the upper incisors, which was diagnosed by orthopantomography. Approximately one year after starting orthodontic treatment, the patient went to the emergency department because of a phlegmonous tumefaction of the lateroposterior upper left maxillary region. Diagnostic orthopantomography and axial computed tomography scan results of the facial skeleton revealed a large lesion occupying the left maxillary sinus, rhizolysis of dental elements 26 and 27, and dislocation of dental element 28. The lesion and infected sinus mucosa were removed through surgical antral-cystectomy with the Caldwell-Luc approach. Histological examination of the lesion confirmed the suspected diagnosis of keratocystic odontogenic tumor. The 12-month follow-up orthopantomography and computed tomography scan results showed good trabecular bone formation in the lesion area. The 24-month follow-up results showed optimal healing in the area of the lesion, positive pulp vitality tests for teeth 26 and 27, and good periodontal tissue healing, as verified through periodontal probing.

**Conclusions:**

Combined with our observations from a careful review of the literature, the results of the case study suggest that keratocystic odontogenic tumor and dental agenesis probably do not develop through a common genetic cause. More likely, they are caused by related environmental factors. Management of this case required the multidisciplinary collaboration of different specializations and careful planning to devise a correct therapeutic protocol and reach a favorable prognosis.

## Introduction

The classification of maxillary cysts by the World Health Organization (WHO) in 1992 categorized keratocysts as maxillary cysts of dysembryonic odontogenic origin. In 2005, the WHO defined keratocysts as benign, uni- or multi-cystic intraosseous neoplasms of odontogenic origin (for example, arising from epithelial residues of the dental lamina). Keratocysts typically have a thick, parakeratinized, stratified, squamous epithelial lining, and a potentially aggressive and infiltrating behavior [1-3]. The well-known aggressive evolution of keratocysts, their histology, and new findings in genetics led the WHO (year 2005) to reclassify these lesions as keratocystic odontogenic tumors (KCOTs) [3,4].

KCOTs can develop in single or multiple form. Multiple keratocysts are characteristic of Gorlin-Goltz or nevoid basal cell carcinoma syndrome. Single keratocysts have a frequency of 5 to 15 percent, compared to 5 percent for multiple keratocysts. The frequency of recurrence ranges from 25 to 30 percent for single keratocysts, and is higher for multiple keratocysts. The likelihood of recurrence is currently predicted on the basis of histology; recurrence is rarely associated with orthokeratosis, but frequently found in parakeratosis. KCOTs have equal prevalence in males and females. They can affect individuals of all ages, with peaks in the second and fourth decades of life. They are more common in the posterior mandible (65 to 70 percent of cases), especially in the posterior body and ascending ramus [5,6].

Clinically, a keratocyst manifests as a fast-growing aching tumefaction, with infiltration and expansion in the cortical bone. It may cause rhizolysis and dental dislocation. On radiography, a keratocyst appears as a uni- or multi-locular radiotransparency. A unilocular keratocyst can be connected to the tooth apex, simulating a periapical cyst. Alternatively, it can surround the crown of an impacted tooth, potentially being confused with a dentigerous cyst. If the keratocyst is localized between tooth roots, it may be misdiagnosed as a lateral periodontal or lateral radicular cyst. A KCOT that develops along the midline simulates a nasopalatine duct cyst. Unilocular keratocysts and ameloblastomas are indistinguishable on X-ray [6].

The aim of this report was to study the etiopathogenetic and clinical manifestations of KCOTs, particularly in association with dental anomalies of number. The overall goal was to provide useful information for the correct diagnosis, treatment, and prognosis of these lesions, within a multidisciplinary approach [7-9].

## Case presentation

**Figure 1:**
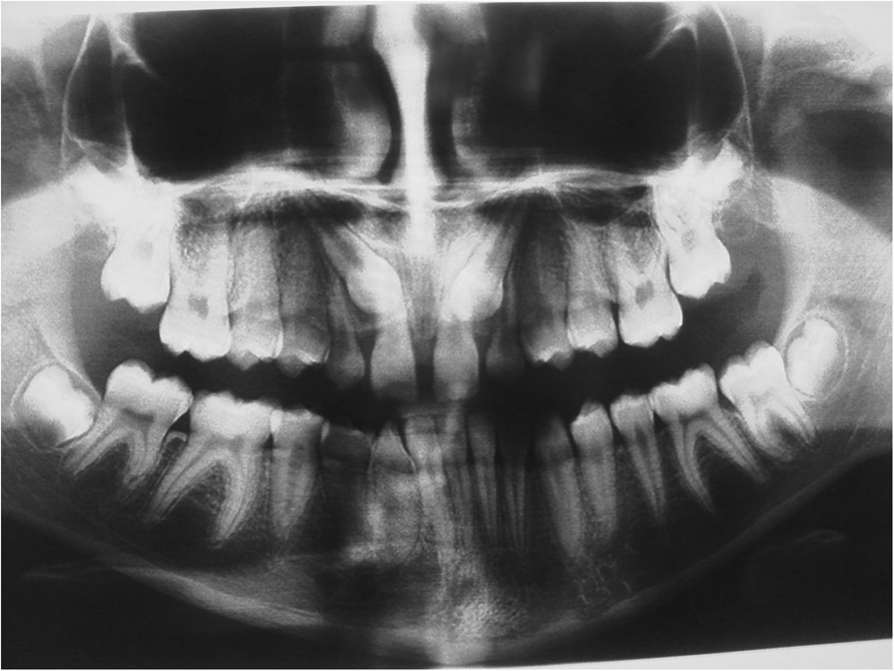
Orthopantomography with agenesis of 1.2 and 2.2.

**Figure 2:**
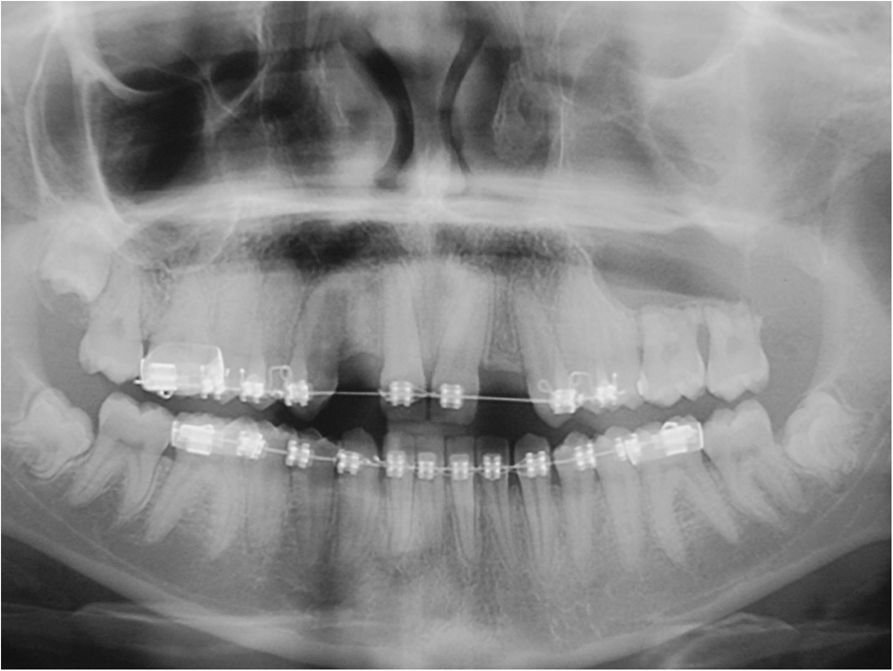
Dental panoramic radiograph.

Here, we report the case of a 14-year-old Caucasian girl, who presented with bilateral agenesis of the upper incisors, diagnosed via orthopantomography (OPG) (Figure 1) and skull X-ray in the laterolateral projection. Approximately one year after starting orthodontic treatment, our patient came to the emergency department because of a phlegmonous tumefaction of the lateroposterior upper left maxillary region. A careful intraoral examination revealed remarkable swelling and mobility of teeth 26 and 27, which were positive to vitality tests. OPG (Figure 2) revealed a large area of unilocular osteolysis, with a calcific peripheral edge in the left maxillary sinus; radicular resorption of teeth 26 and 27; and the absence of tooth 28, which had been present in the previous radiographic examination (Figure 1).

**Figure 3:**
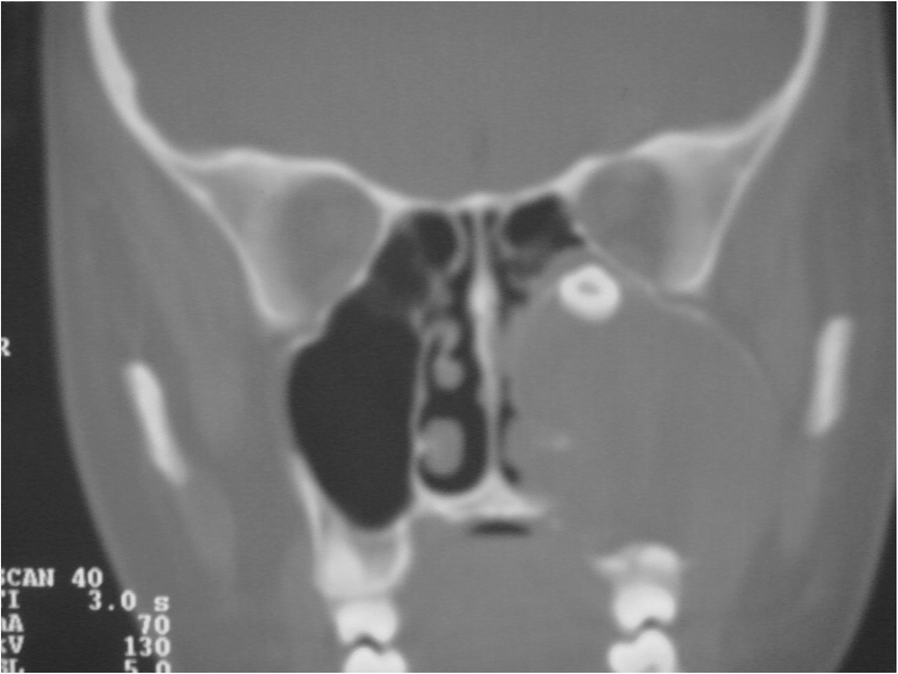
Computed tomography scan: coronal sections.

**Figure 4:**
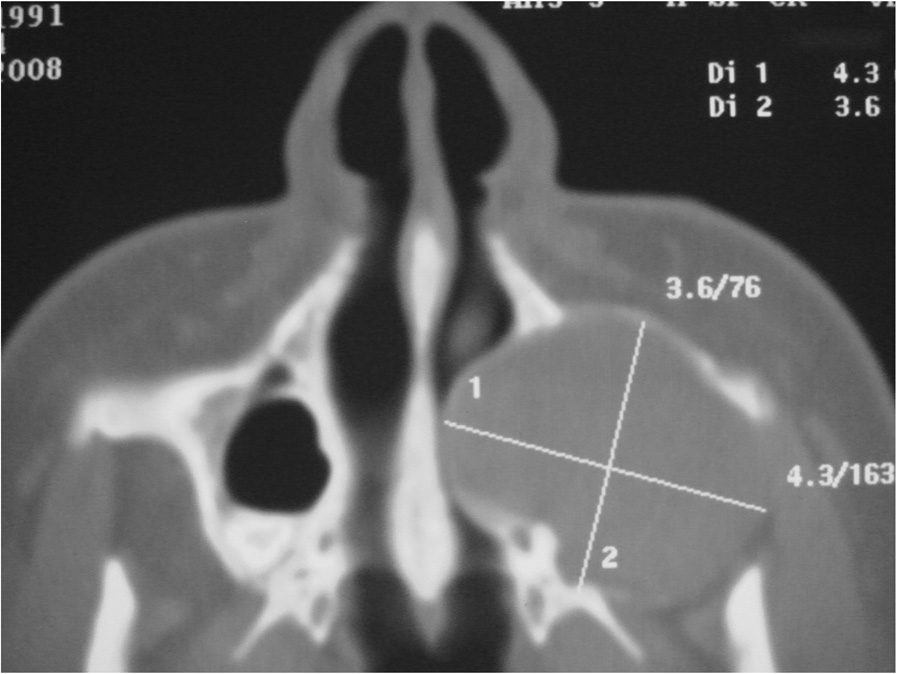
Computed tomography scan: axial sections.

To determine the origin of the lesion and the best surgical approach, an axial computed tomography (CT) scan of the facial skeleton was requested. The coronal section showed complete opacity of the left maxillary sinus, involvement of ethmoidal cells (Figure 3), and dislocation of tooth 2.8 in the sinus. The axial projection showed a large, oval-shaped area of osteolysis, measuring 3.6 × 4.3mm, with deformation and erosion of the buccal cortical bone (Figure 4). Considering this erosion, we immediately performed a needle biopsy of the lesion. The lesion was hemorrhagic and purulent, typical of an infected cyst. After a careful evaluation, we chose antral-cystectomy with the Caldwell-Luc technique, under general anesthesia, as the treatment for removal of the lesion and the infected sinus mucosa.

**Figure 5:**
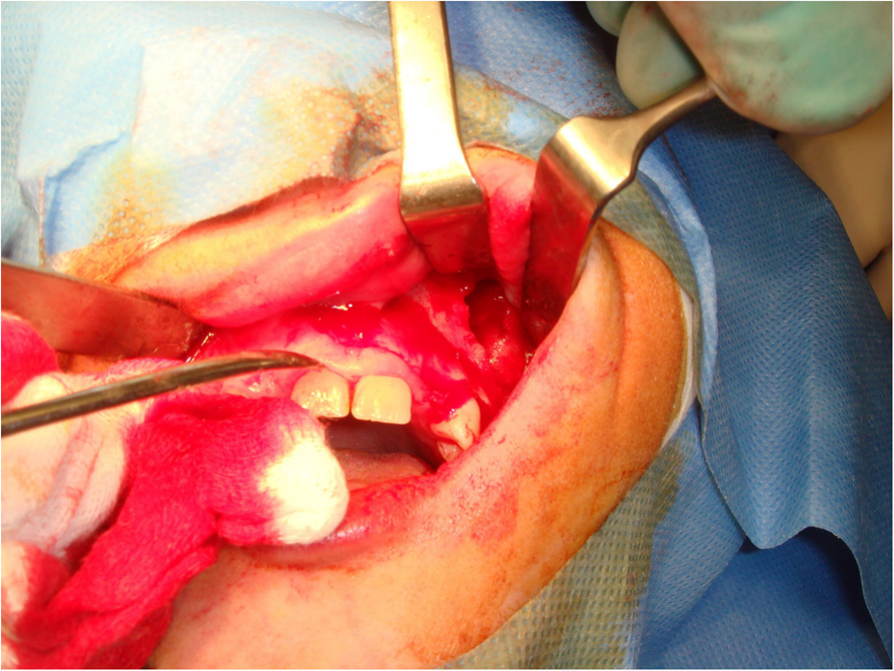
Opening of the flap.

**Figure 6:**
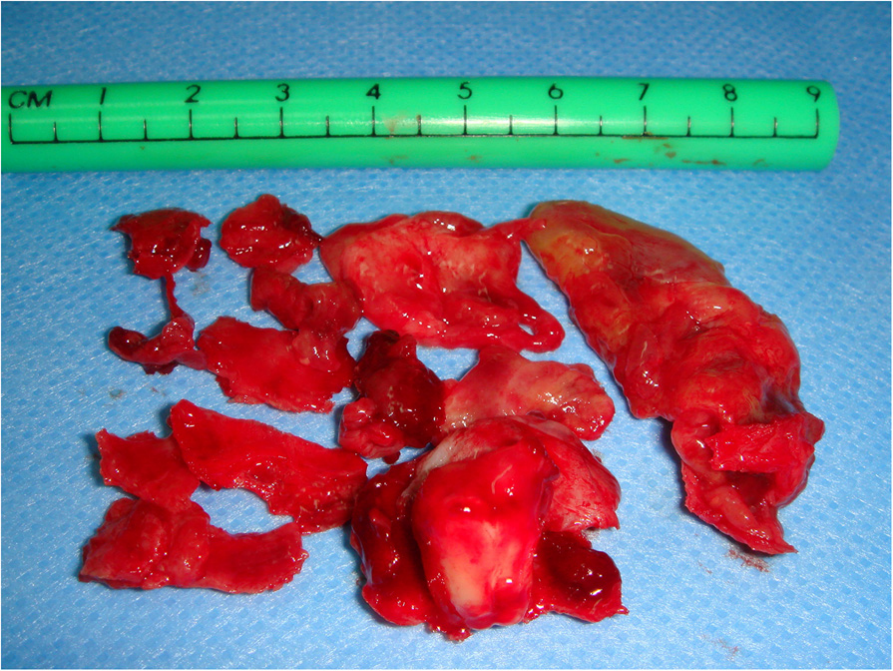
Macroscopic findings.

A large mucoperiosteal incision was made to create a mixed flap (marginal-paramarginal) from the central contralateral incisor to the maxillary tuberosity (Figure 5). Removing the flap revealed a large opening in the cortical bone. The maxillary sinus was emptied through complete enucleation of the keratocyst and removal of the ethmoidal membrane, including the eighth. The removed lesion (Figure 6) was immersed in 10 percent formalin and sent to a pathologist for histological examination. Teeth affected by the lesion were stabilized by interocclusal splinting with wrought metal wire. A histological diagnosis of ‘odontogenic keratocyst with a specific flogistic and chronic focus’ confirmed the clinical suspicion.

**Figure 7:**
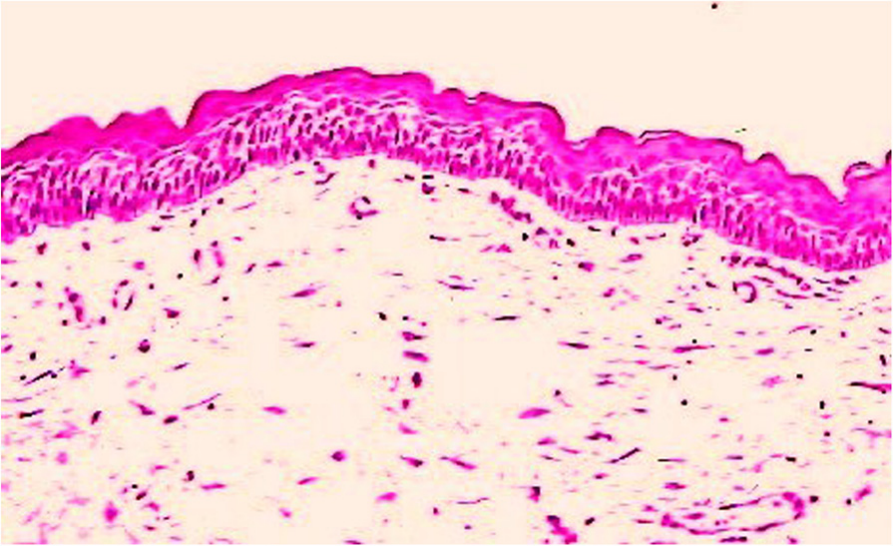
Histological findings.

**Figure 8:**
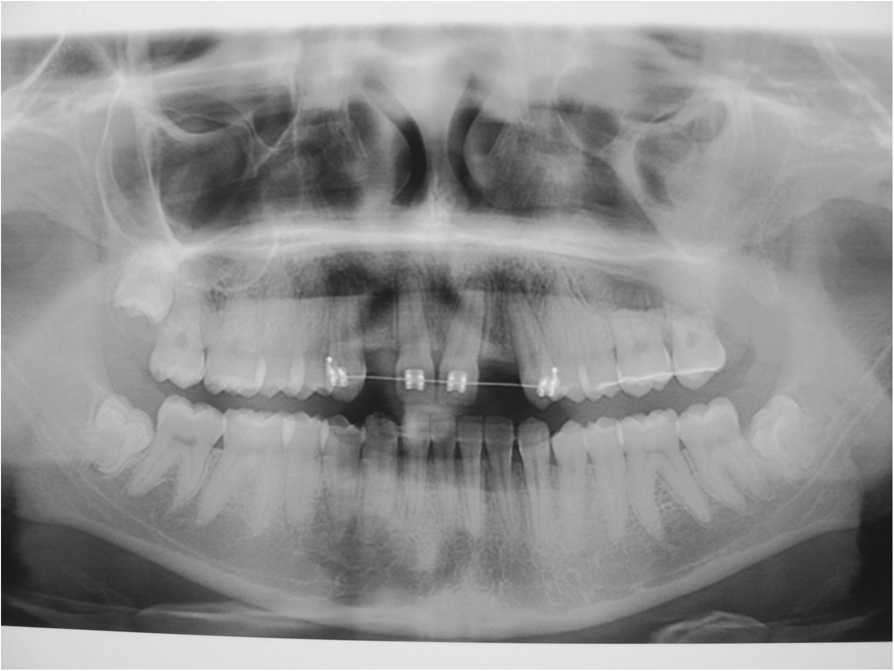
Orthopantomography follow-up after 6 months.

**Figure 9:**
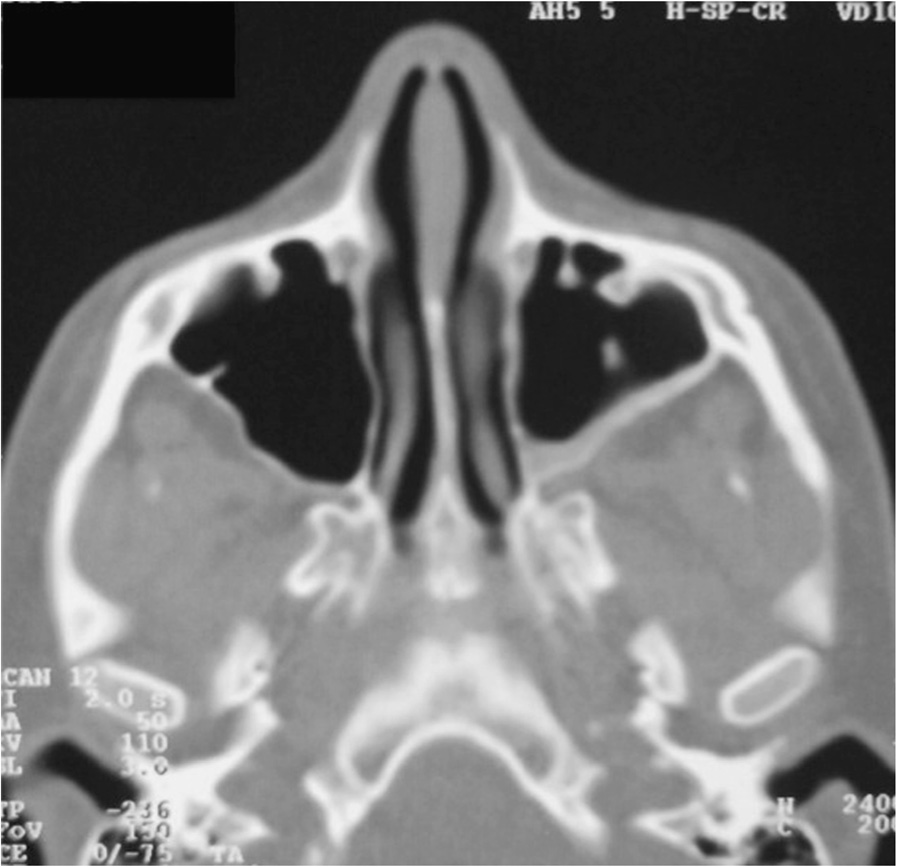
Computed tomography axial projection at 12 months.

**Figure 10:**
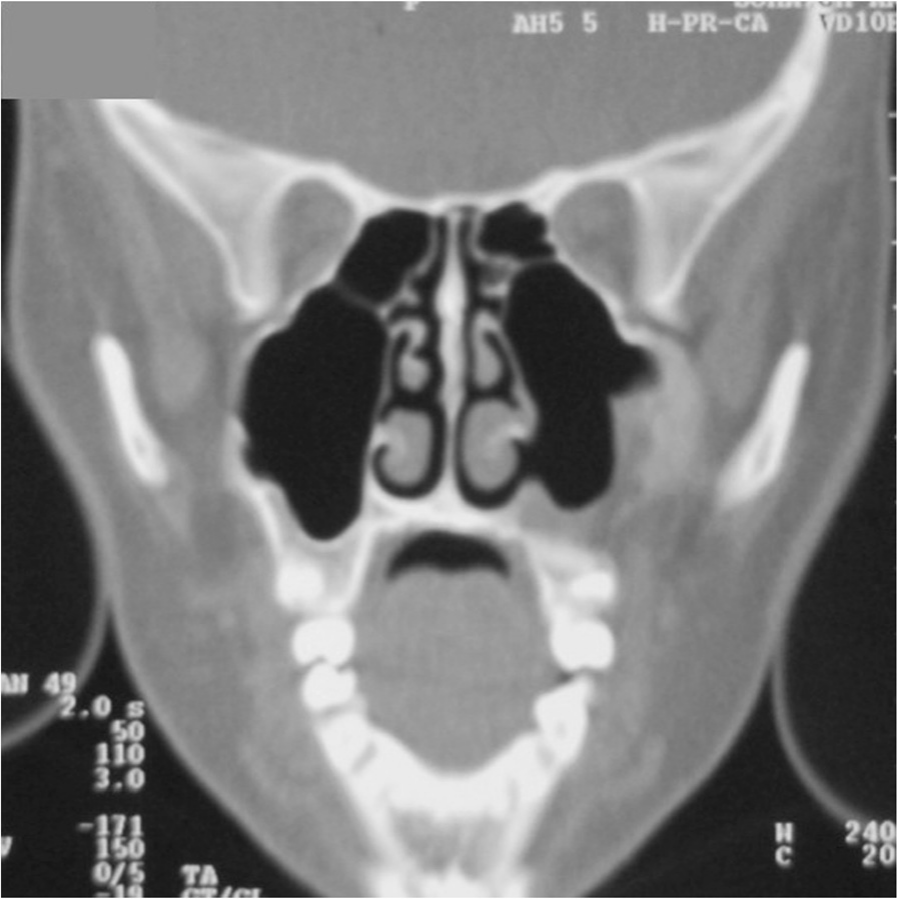
Computed tomography coronal projection at 12 months.

**Figure 11:**
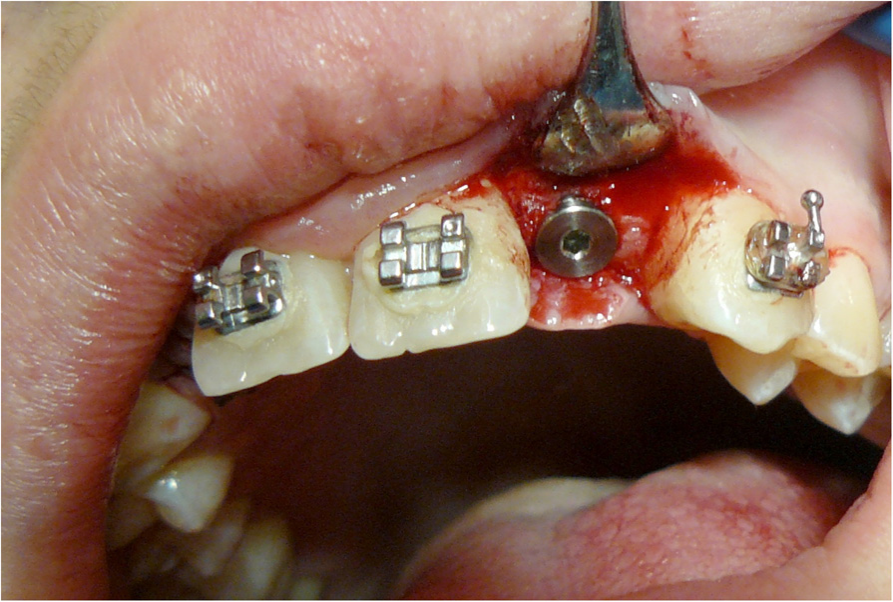
Details of implant on 2.2.

The KCOT showed a typical undulated pattern in the epithelium, accompanied by parakeratinization and exfoliation of keratin in the lumen (Figure 7). Clinical and imaging follow-ups at 6, 12, and 24 months showed good bone trabeculation in the area of the KCOT (Figures 8, 9 and 10), and positive pulp vitality results for teeth 2.6 and 2.7. Probing indicated healing of the periodontal area. Teeth neighboring the missing teeth were subjected to radicular torque, and internal hexagon implants (3.5 and 4mm in diameter) were inserted (Figure 11). Temporary rehabilitation was performed. Loading was deferred for 30 days after implant insertion (Figure 11).

**Figure 12:**
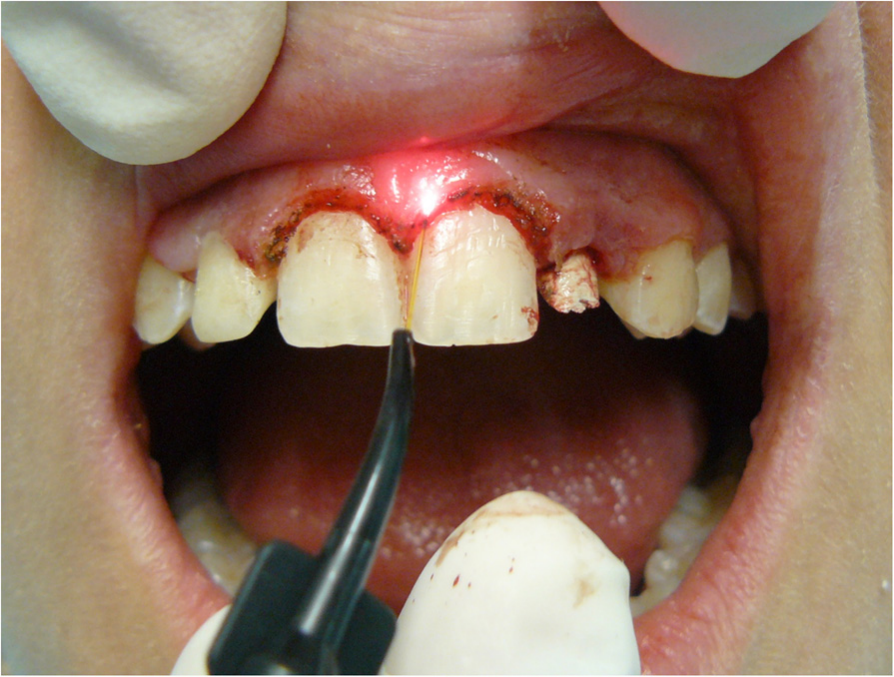
Gingivectomy with Nd:YAG laser.

The orthodontic braces were removed, resulting in an increased gum volume and ‘gummy smile’. Therefore, we performed gingivectomy using an Nd: YAG laser source (Figure 12).

**Figure 13:**
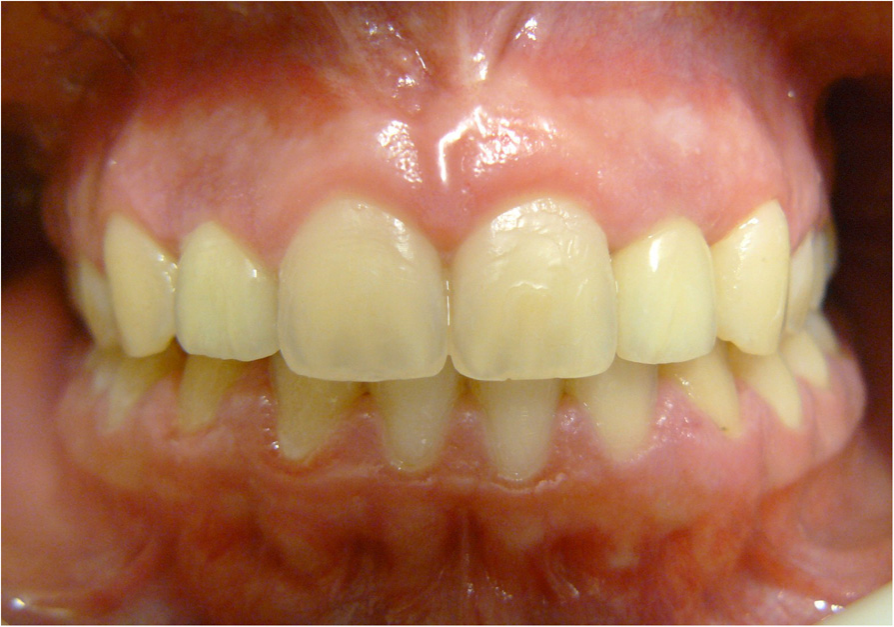
Completed case.

**Figure 14:**
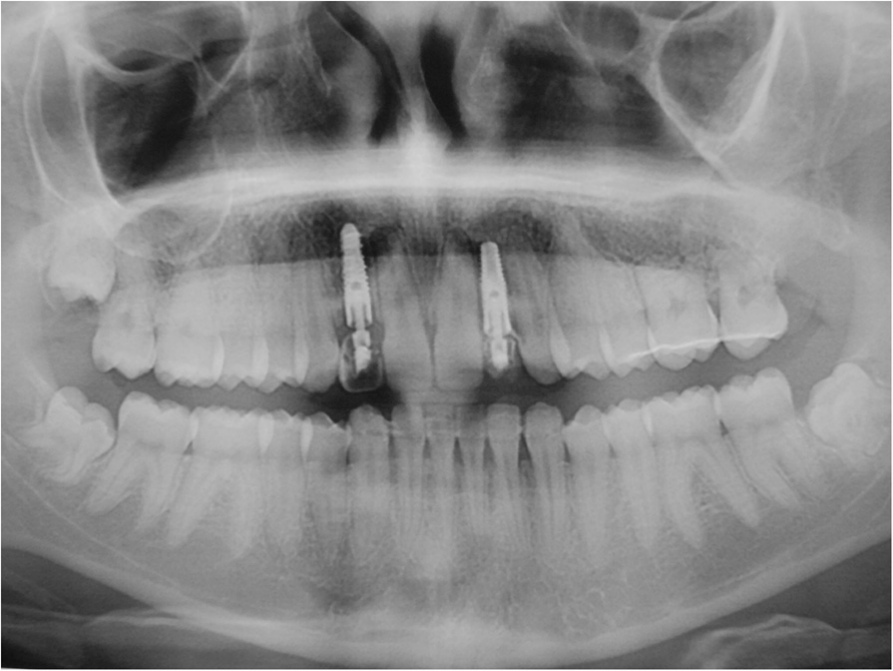
Orthopantomography examination at 24 months.

Sixty days after implant insertion, the peri-implant soft tissues were determined to be in an excellent condition, and the final prosthetic rehabilitation with a ceramic implant-crown system was performed (Figure 13). At the 24-month follow-up visit, there was no recurrence, and the vitality of the dental elements was confirmed (Figure 14).

## Discussion

In this manuscript, we present the case of a patient with a KCOT, an infrequent pathology of the maxillary sinus, associated with a dental anomaly (hypodontia). We primarily focused on the etiopathogenesis of the lesion, to detect a possible causal relationship between KCOT and agenesis.

### Keratocyst: malformation or neoplasm?

No predisposing factors for keratocysts are known. Like other odontogenic tumors, keratocysts usually appear *de novo*. In rare cases, they develop on odontogenic cysts. Approximately 200 genes are involved in tooth development. Among them, fibroblast growth factor (*FGF*) and sonic hedgehog (*SHH*) are the most frequently altered genes in odontogenic neoplasms.

The question of whether keratocysts are malformations or neoplasms has been the subject of long debate. Whereas some authors claim that a keratocyst develops from a distal extension of the dental lamina, others think it is derived from the basal layer of the oral epithelium. Most authors agree that the neoformation shows an expanding growth type, similar to maxillary cysts [10,11]. Keratocysts have an actively proliferating epithelium. They exhibit high numbers of cells positive for Ki67, proliferating cell nuclear antigen (PCNA), and p53 compared to other odontogenic cysts [12]. PCNA and Ki67 are proliferation markers that are expressed during mitosis. The oncosuppressor p53 favors cell apoptosis, and is frequently mutated or overexpressed in neoplasms. From these observations, we can deduce that B-cell lymphoma-2 (Bcl-2), matrix metalloproteinase (MMP)-2 and -9, transforming growth factor (TGF), and interleukin (IL)-1a and -6 are overexpressed in keratocysts.

Patched (PTCH) is the receptor for secreted sonic hedgehog (SHH) protein. Normally, PTCH forms a receptor complex with the product of the oncogene Smoothened (*SMO*), through the SHH ligand. The interaction between PTCH and *SMO* inhibits translation of the growth signal. If the normal functioning of PTCH fails, then the proliferation-promoting effects of *SMO* become dominant [7,9]. *SMO* is an oncosuppressor that is important for the embryonic development of neural tubes, skeleton, limbs, lungs, hair follicles, and teeth. Mutations of *SMO* can be found in keratocysts in Gorlin-Goltz syndrome, as well as in sporadic keratocysts. The above findings constitute clinical and molecular evidence for the conclusion that keratocysts are neoplasms characterized by local aggressiveness (see [3]), and should be referred to as ‘keratocystic odontogenic tumors’ [1-3,13,14].

### Dental agenesis: genetic and/or environmental etiology

Concerning the etiopathogenesis of dental agenesis, the hereditary theory (Brook [15]; Magnusson [16]; Graber [17]; Ahmed *et al.*[18]) highlights a dominant autosomal transmission, with reduced penetrance and variable expression in terms of the number and region of the missing teeth. Genetic surveys of members of families with dominant autosomal oligodontia highlighted mutations in two transcription factors: Msh homeobox 1 (*MSX1*) in chromosome 4 (4p.16), and Paired box gene 9 (*PAX9*) in chromosome 14 (14q.21q.13) [15-18]. Functional deletion of these genes in rats arrested tooth development and caused tissue alterations [19,20]. Other recent studies suggested a possible relationship of agenesis with deletion of the gene TGF alpha (*TGFA*) [20].

Some authors have suggested that a recessive autosomal transmission may be possible (Grahnen [21]; Ahmad [22]). Suarez and Spence [23] stated that agenesis may result from a polygenic or multifactorial heredity model, rather than from a single dominant gene. Brook [24] supported a multifactorial model, in which polygenic factors played a fundamental role, but without excluding environmental factors [25].

Studies agree on the importance of pathology during the maturation and migration processes of cells involved in dental gem formation (that is, after 120 embryonic days, for the permanent dental elements). Environmental factors can prevent follicle formation, due to internal factors (for example, pathologies of the hypophysis, thyroid, or parathyroid, and so on) or external factors (for example, maxillary trauma or surgery, early infection, serious nutritional insufficiency, radiotherapy, and so on) [19,26]. Arrest of dental development has been attributed to environmental factors, on a background of genetic predisposition to agenesis (Schalk Van Der Weide) [27-29]. Thus, KCOT and dental agenesis are likely not due to a common genetic cause, but are probably related to casual environmental factors.

### Case study of KCOT with dental agenesis

The present study concentrated on clinical, imaging, and histological diagnosis, as well as on the therapy of the lesion and agenesis. Initially, healing the KCOT was the most important concern. We applied an aggressive approach (that is, enucleation of the lesion with maxillary antrostomy, using the Caldwell-Luc technique), which prevented recurrence and encouraged bone repair. From a radiological perspective, this approach led to a progressive increase in trabecular bone formation during later follow-ups.

The final phase of the intervention concentrated on the bilateral agenesis of the upper incisors through an implant-supported prosthetic treatment. From the biological, functional, and esthetic perspectives, implant therapy was considered to be the most desirable treatment. Prior to implant insertion, orthodontic treatment was used to achieve and maintain the space necessary for the implant-supported prosthetic reconstruction.

## Conclusions

The management of this case required multidisciplinary collaboration between different specializations. It necessitated careful planning to devise the ideal therapeutic protocol for a favorable prognosis, including absence of KCOT recurrence. Good functionality and esthetics were obtained through the implant-supported prosthetic rehabilitation of dental agenesis.

## Consent

Written informed consent was obtained from the patient’s legal guardian for publication of this case report and any accompanying images. A copy of the written consent is available for review by the Editor-in-Chief of this journal.

## Abbreviations

CT: computed tomography; KCOT: keratocystic odontogenic tumor; OPG: orthopantomography.

## Competing interests

All authors declare that they have no competing interests.

## Authors’ contributions

ML and VL contributed to the conception and design of the study, the analysis and interpretation of the data, and drafted the manuscript. ML, VL, and MC made the diagnosis of KCOT and established the treatment. AM, GM, and MC were involved in the data interpretation, and contributed to the revision of the drafted manuscript. All authors read and approved the final manuscript.
